# Exercise-Induced Hypertension in Healthy Individuals and Athletes: Is it an Alarming Sign?

**DOI:** 10.7759/cureus.11988

**Published:** 2020-12-09

**Authors:** Linha (Lina) M Mohammed, Meera Dhavale, Mohamed K. Abdelaal, A B M Nasibul Alam, Tatjana Blazin, Dhruvil Prajapati, Jihan A Mostafa

**Affiliations:** 1 Miscellaneous, California Institute of Behavioral Neurosciences & Psychology, Fairfield, USA; 2 Internal Medicine, California Institute of Behavioral Neurosciences & Psychology, Fairfield, USA; 3 Surgery/General and Visceral Surgery, California Institute of Behavioral Neurosciences & Psychology, Fairfield, USA; 4 Psychiatry/Psychotherapy, California Institute of Behavorial Neurosciences & Psychology, Fairfield, USA

**Keywords:** cardiovascular risk, exercise, athletes, hypertension, blood pressure, sports medicine, prevention of cardiovascular disease

## Abstract

Exercise-induced hypertension (EIH) is defined as elevated blood pressure (BP) > 190mm Hg for females and > 210 mmHg for males during exercise. EIH is prevalent among athletes and healthy individuals with no cardiovascular (CV) risk factors. While previous data corroborates exercise in reducing hypertension and cardiovascular risk, the development of EIH and its attendant cardiovascular risk necessitates a review of the pathophysiological mechanisms resulting in EIH. To date, these mechanisms causing EIH are not fully understood, nor are there any established guidelines on the management of EIH. In this article, we discuss in detail the pathophysiological mechanisms, the prognostic value, clinical implications, possible treatment, and future directions in managing EIH.

## Introduction and background

Hypertension has become the most prevalent cardiovascular risk factor among the U.S. population and worldwide [1]. It has been estimated that 45% of the U.S. population suffers from hypertension, and nearly one out of two adults >18 years old in the United States have hypertension [2]. The role of exercise and physical activity for preventing chronic diseases (e.g., cardiovascular disease, diabetes, and hypertension) has been well described [3,4]. Even though hypertension is commonly a disease of older individuals, young athletes and active individuals have shown a higher prevalence of hypertension than generally appreciated [5]. 

Exercise-induced hypertension (EIH) is one factor that could be linked to the development of hypertension in young athletes and healthy individuals later in their lives [6]. EIH is defined as a systolic BP of > 190 mmHg for females and >210 mmHg for males in individuals undergoing exercise stress testing [7]. Yzaguirre I, et al. reported that a systolic BP of > 180 mmHg at moderate-intensity exercise or diastolic BP of > 95mmHg at maximum exercise was the best predictor to develop late-onset hypertension in healthy young individuals [8]. 

Previously, data regarding the prognostic value of high BP response to exercise in athletes has been conflicting. Some studies reported that hypertension in response to exercise had limited prognostic value [9]. Other studies, however, reported that EIH is linked to the future development of hypertension [10,11]. Currently, it has been determined that EIH is an independent risk factor for cardiovascular events and mortality, left ventricular (LV) hypertrophy, and cardiac injury, leading to arrhythmias, atrial fibrillation, myocardial fibrosis, and sudden cardiac death [12,13]. Interestingly, a very recent study has shown that EIH may be associated with a 3.6-fold increased risk of hypertension in highly trained athletes [6]. Despite all recent data on the increased risk of cardiovascular (CV) events and mortality associated with EIH, the pathophysiology and the management EIH has been less appreciated [14-16].

With the increasing concern that high blood pressure during exercise should be a warning signal to healthy young adults and athletes on developing hypertension and cardiac injury, early intervention is important to reduce the risk of end-organ damage [8]. Therefore, in this review, we will examine the pathophysiological mechanism behind exercise hypertension, and understand the prognostic value, clinical implication, and the best possible management.

## Review

The normal physiological response of BP during exercise

Normally, blood pressure rises during exercise due to increased cardiovascular demand and oxygen uptake from working muscles [17]. There is an immediate increase in sympathetic activity and heart rate to boost cardiac output [18]. With the increase in cardiac output and stroke volume, there is an active increase in compliance and vasodilatory effect of the arteries of the skeletal muscles and tissues to accommodate an increase in blood flow (Poiseuille’s Law); thus, there is an increase in arterial pulse pressure [19].

Pathophysiology of EIH

*Role of Renin-Angiotensin-Aldosterone System* *(RAAS) on EIH*

The pathophysiologic mechanism of EIH is still unclear. Several studies have studied the pathophysiological response that leads to EIH. It has been found that EIH is linked to angiotensin II and nitric oxide (NO) activities during exercise. The renin-angiotensin-aldosterone system (RAAS) changes during and after exercise treadmill test in marathon runners were observed by Chul-Hyun Kim [20]. The study reported a reduction in angiotensin II and no significant change in NO in people with normal blood pressure after exercise. However, angiotensin II remained elevated with a reduction in NO in runners with EIH. Since the RAAS system plays a role in regulating blood pressure, these results may explain the reduction in the vasodilatory effect of arteries, leading to an increase in BP during exercise [20].

Endothelial Dysfunction 

Proper physical activity is well known to have a positive effect on vascular endothelial function. One mechanism by which physical activity improves endothelial function is the activation of endothelial NO synthase (eNOS) enzyme expression. This is essential in reducing oxidative stress and the reactive oxygen species (ROS) generated by working muscles. This mechanism is also important to increase nitric oxide (NO) bioavailability, thus, improving coronary blood flow and decrease vascular resistance [21-23]. Tzemos N examined the endothelium vascular function during exercise in healthy young individuals with no cardiovascular risk factors [24]. The study concluded that exposure to high BP during exercise affects endothelial function by reducing nitric oxide (NO) release and arterial stiffness [24].

Mechanism of Arterial Stiffness

One of the earliest studies to evaluate the effect of regular, intense exercise on arterial elasticity was by Vlachopoulos C, et al. [25], who measured aortic stiffness with carotid-femoral pulse velocity (PWV) in 49 marathon runners. The study found higher PWV (P < 0.01) and higher pulse pressure in marathon runners than the active, healthy control group [25]. The potential mechanisms that could lead to increased arterial stiffness in athletes are repetitive, chronic high pressure on the arterial wall potentially leading to arterial fibrosis secondary to its effect on the intramural elastic elements [26,27]. The second possible mechanism is the sympathetic activation resulting in reduced arterial compliance and decreased vascular compliance [25,26, 28,29].

Clinical Implications

EIH and Cardiovascular Risk

Several studies show consistency in reporting a significant correlation between EIH and cardiovascular risk and function [11,16,30-33]. The most commonly reported findings were an increase in LV mass and left atrial size in athletes [12]. Longás Tejero et al. reported 75% of young athletes with EIH to have severe LV hypertrophy [34]. The structural change in atrial size can increase arrhythmias risk, particularly atrial fibrillation (Afib). Kim et al. and Abdulla et al. both reported that atrial fibrillation is 2.5 times more common in athletes who perform a high-intensity exercise such as marathon running and five times more common in athletes than the general population [35,36]. Whether these structural heart changes result from physiologic adaptation to exercise or hypertension is uncertain [37]. Theoretically speaking, increased blood pressure and the workload on the left ventricle resulting in LVH is a physiologic adaptation [38]. However, previous studies reported that this physiologic response to exercise was not associated with an increased risk of cardiovascular adverse events (e.g., arrhythmias) seen in hypertension-related LVH [38,39]. Therefore, it may be crucial to identify LVH related specifically to EIH and differentiate hypertension-related LVH to prevent cardiovascular adverse events such as arrhythmias, atherosclerosis, and sudden cardiac death. Studies showing high N-terminal-pro-brain natriuretic peptide (NT-pro-BNP) and troponin-I serum levels suggest that left ventricular dysfunction may be associated with exercise-induced myocardial fatigue and injury. Several studies reporting high levels of cardiac markers (NT-ProBNP and Troponin-I) in marathon runners after exercise suggest that athletes exposed to chronic EIH may suffer an ongoing cardiac injury [6,30,40]. A recent study by the European Journal of Preventive Cardiology demonstrated, using contrast-enhanced cardiac magnetic resonance (CMR), how exercise-induced cardiac injury may be associated with the pathologic remodeling leading to left ventricular pathology. The study showed a marked increase in cardiac markers after exercise is linked to increased volume overload and LV filling pressure. Another finding was that for athletes with elevated cardiac markers (NT-ProBNP and Troponin-T), no myocardial inflammation or edema was detectable after exercise. However, most athletes in this study showed a positive enhancement on CMR, indicating myocardial fibrosis (P <0.01). During exercise, chronic high BP exposure causes a continuous pressure/volume overload, leading to a change in the left atrium( LA), LV normal filling patterns, and the LV diastolic dysfunction resulting in myocardial fibrosis [14,31]. 

Table 1 shows the studies used in this review that reported a significant correlation between EIH and cardiovascular risk.

**Table 1:** Studies included in this review with a significant correlation between EIH and cardiovascular risk EIH: exercise-induced hypertension

Study	Number of Subjects	P-Value	Associated EIH with Cardiovascular Diseases
Exercise-induced arterial hypertension - an independent factor for hypertrophy and a ticking clock for cardiac fatigue or atrial fibrillation in athletes?	51	0.034	Yes
Exercise-induced hypertension can increase the prevalence of coronary artery plaque among middle-aged male marathon runners	50	<0.05	Yes
Hypertensive response to exercise: a potential cause for new wall motion abnormality in the absence of coronary artery disease	548	0.012	Yes
Effects of Marathon Running on Cardiac Markers and Endothelin-1 in EIH Athletes	22	<0.05	Yes
Acute impact of an endurance race on cardiac function and biomarkers of myocardial injury in triathletes with and without myocardial fibrosis	30	<0.01	Yes

Future development of hypertension

Numerous studies have reported a consistent, predictive relationship between EIH and the future development of hypertension [8,17,18,32,41]. Normally, systolic BP is measured during exercise stress tests. Its clinical value, however, has not been appreciated [42]. One of the first and largest meta-analysis studies that investigated the prognostic value of exercise BP in 46,314 individuals reported that high exercise BP during or after moderate exercise increased the risk of cardiocerebrovascular outcome (fatal, nonfatal stroke, coronary artery disease, and myocardial infarction by 36% (95 % CI, 1.02-1.38, P = 0.039) after adjusting the age, gender, and other CV risk factors [7]. Data from recent studies also confirmed high BP induced by moderate-intensity exercise is associated with the early development of hypertension [8,41]. With millions of exercise stress tests are performed worldwide daily [19], high exercise BP measured during exercise tests should be considered a warning sign. Individuals with high BP during exercise should be monitored closely using an ambulatory clinic BP sphygmomanometer to detect hypertension and prevent end-organ damage.

Treatment

Previous studies confirmed EIH is a risk factor for the development of hypertension and cardiocerebrovascular disease. However, screening and treating EIH is still controversial. Presently, treatment guidelines for EIH are still not included in the current recommended guideline to manage athletes' hypertension. As discussed previously, the most common pathophysiological mechanisms behind EIH are elevated angiotensin II, reduction in nitric oxide (NO) levels, and an increase in sympathetic tone. Thus, angiotensin-converting enzyme (ACE) inhibitors and B-blockers would be the best possible treatment options [17,20]. Antioxidant supplements were also considered a great measure to reduce free radicals and oxidative stress, resulting from excessive training, therefore maintaining vascular endothelial health and function [21]. Most athletes exercise vigorously for a duration (90-300 min/day) 10 times higher than the recommended duration given by the American College of Sports Medicine and the American Heart Association [43]. Therefore, a reduction in the duration and the intensity of exercise may be warranted to prevent cardiovascular adverse events [35].

Limitations

Some of the selected studies were performed on a small sample population, impacting the study's power. Since this article is a traditional review, we did not perform a quality assessment of the selected studies.

## Conclusions

EIH is prevalent among athletes and healthy individuals with no cardiovascular risk factors. EIH causes cardiac remodeling, left ventricular dysfunction, induces myocardial injury and fibrosis, increased risk for cardiovascular events and mortality (atherosclerosis, arrhythmias), and future hypertension development. Previous studies defined EIH is as a systolic BP of > 190 mmHg for females and >210 mmHg for males during exercise. An increase in sympathetic tone resulting in elevated levels of angiotensin II seems to be the common mechanism behind EIH. Also, endothelial dysfunction and an increase in arterial stiffness may be underlying pathophysiological mechanisms of EIH. Given the prognostic significance of EIH in our study, we recommend conducting further studies to fully understand the type and the level of exercise intensity associated with increased risk of cardiovascular events and future development of hypertension. We also consider using exercise tests as a pre-screening tool to screen for elevated BP during exercise. Those with high BP should be closely monitored using an ambulatory clinic sphygmomanometer to prevent future hypertension and end-organ damage.
